# Validation and reliability of the Athens Insomnia Scale-8 in Chinese psychiatric inpatients

**DOI:** 10.3389/fpsyt.2026.1651778

**Published:** 2026-05-08

**Authors:** Wei Li, Yi-Yuan Qiao, Jie Zhong

**Affiliations:** 1 Shanxi Bethune Hospital, Shanxi Academy of Medical Sciences, Third Hospital of Shanxi Medical University, Tongji Shanxi Hospital, Taiyuan, China; 2 Beijing Key Laboratory of Behavior and Mental Health, Clinical and Health Psychology Department, School of Psychological and Cognitive Sciences, Peking University, Beijing, China

**Keywords:** AIS-8, insomnia assessment, measurement invariance, reliability, validity

## Abstract

**Background:**

Existing cross-cultural applications of the Athens Insomnia Scale-8 (AIS-8) have been constrained by sample limitations, most notably the absence of psychiatric inpatients in validation datasets. This gap underscores the need to contextualize the scale’s performance within specialized clinical populations. Accordingly, this study aims to systematically assess the validity and reliability of the AIS-8 and characterize its psychometric properties among a sample of Chinese psychiatric inpatients, addressing an unmet critical need in cross-cultural insomnia assessment research.

**Methods:**

A total of 519 psychiatric inpatients completed the Chinese version of the AIS-8, along with the Generalized Anxiety Disorder-7 (GAD-7) and Patient Health Questionnaire (PHQ-9). After excluding missing values, 411 participants were included in the final analysis.

**Results:**

Exploratory factor analysis (EFA) and confirmatory factor analysis (CFA) revealed a two-factor model structure of “nighttime sleep problems” and “daytime dysfunction” for the AIS-8 in Chinese psychiatric populations. Although the item “daytime sleepiness” showed relatively low factor loadings, model fit deteriorated when this item was removed. Strict measurement invariance was established for gender, whereas age, marital status, and occupation exhibited only weak invariance. The AIS-8 demonstrated a Cronbach’s alpha of 0.84, and convergent validity was supported by significant positive correlations with the PHQ-9 and GAD-7.

**Conclusion:**

The Athens Insomnia Scale-8 (AIS-8) demonstrated acceptable reliability and validity in a sample of Chinese psychiatric inpatients, providing preliminary support for filling an important gap in its cross-cultural validation among specialized psychiatric populations. Given the study’s geographical scope and sample characteristics, which represent inherent limitations, the findings tentatively suggest that the AIS-8 may be used as a reliable instrument for clinical insomnia assessment in Chinese psychiatric settings.

## 1 Introduction

Insomnia, characterized by difficulty falling asleep or maintaining sleep, has emerged as a global public health concern affecting at least one-third of the population (1–3). This sleep disorder frequently co-occurs with mental health conditions such as anxiety and depression, demonstrating a bidirectional relationship (3–5). Previous studies have shown positive correlations between insomnia and measures of general anxiety and major depression (6–10).

In light of the profound ramifications of insomnia on physiological and psychological well-being alongside daily functionality, the detection, thorough evaluation, and management of insomnia constitute critical clinical priorities. The Athens Insomnia Scale (AIS), formulated in consonance with the diagnostic benchmarks of the International Classification of Diseases, 10th Revision (ICD-10) (11), stands as a validated self-administered instrument engineered to quantify the severity of insomnia. Empirical investigations (12) have illustrated that the AIS demonstrates heightened discriminative efficacy in the assessment of insomnia when juxtaposed with the Pittsburgh Sleep Quality Index (PSQI) and Epworth Sleepiness Scale (ESS), primarily attributable to its targeted appraisal of cardinal insomnia manifestations, encompassing sleep initiation latency, sleep maintenance disturbance, early-morning awakening, and concomitant diurnal impairments.

This preeminence may be ascribed to the AIS’s explicit congruence with clinical diagnostic criteria, thereby enabling more meticulous measurement of insomnia severity. In contradistinction to the PSQI, which offers a holistic evaluation of sleep quality, and the ESS, which predominantly gauges pathological diurnal somnolence, the AIS zeroes in on the specific symptomatology of insomnia. Its standardized scoring paradigm and structure facilitate both clinical implementation and scholarly inquiry, thereby augmenting its utility in epidemiological research and interventional trials. It is noteworthy that although the AIS exhibits robust psychometric attributes, it is advisable to integrate this scale with comprehensive clinical assessments and patient case histories to ensure accurate diagnostic interpretation, as no singular tool can entirely supplant the multidisciplinary evaluation requisite in sleep medicine.

Over the past two decades, this questionnaire has been employed not merely for exploring clinical insomnia diagnostics (13, 14) but also extensively validated for its reliability and validity across transnational boundaries in heterogeneous populations (7, 15–17). Notwithstanding, the psychometric characteristics of the AIS manifest divergent attributes across diverse cultural milieus and sample demographics. The original AIS elicited a unifactorial construct comprising 8 items (11), whereas subsequent investigations have denoted multifactor solutions for this scale. Specifically, factor analytic endeavors in Chinese (13), Japanese (14), and Mexican (7) cohorts have segregated the 8 items into two factors: “nocturnal sleep disturbances” (items 1–5) and “diurnal functional impairments” (items 6–8). Conversely, a factor analysis among Turkish populations (18) proposed a two-factor model, albeit with content discrepancies from preceding frameworks—emphasizing “insomnia symptomatology” (items 1–4) and “insomnia-related quality of life sequelae” (items 5–8). It is noteworthy that the two-factor model has demonstrated circumscribed generalizability: Spanish samples (19) documented Heywood cases during model specification, intimating constraints in the two-factor model’s utility. Even within occupational computer user cohorts, a three-factor model has been empirically interrogated, further accentuating the taxonomical variability of the AIS construct. Such divergent modeling outcomes are plausibly attributable to regional heterogeneities and sampling idiosyncrasies.

Within the Chinese research paradigm, the AIS has undergone validation in relatively circumscribed clinical outpatient, nonclinical, normal adolescent (20), and advanced oncological patient samples (21). Significantly, the psychometric adequacy of the AIS among psychiatric inpatients remains underexplored. Moreover, a conspicuous lacuna persists in the global scholarly corpus: paucity of investigations leveraging inpatient psychiatric samples for evaluating the AIS model’s reliability and validity represents a critical frontier for future scientific inquiry.

Moreover, evidence regarding measurement invariance is of paramount importance, as it allows for the verification that AIS scores retain consistent interpretability across subsamples, notwithstanding demographic disparities. While prior research has predominantly employed gender as a metric for assessing invariance, other demographic variables—including occupation, age, and marital status—warrant equal consideration. Previous studies have demonstrated measurement invariance of AIS across genders in international populations (7, 17), but its applicability to age, marital status, and occupational subgroups remains underinvestigated, particularly in psychiatric settings.

In the present investigation, we undertook a systematic evaluation of measurement invariance across gender, age, and occupational categories within a Chinese inpatient psychiatric cohort, with the explicit objective of establishing the cross-demographic validity of the Athens Insomnia Scale (AIS) in the context of Chinese psychiatric clinical practice. Furthermore, we hypothesized that AIS-8 would exhibit a two-factor structure in Chinese psychiatric inpatients and demonstrate measurement invariance across gender, age, marital status, and occupation.

## 2 Methods

### 2.1 Participants and procedures

This study was conducted from October 2023 to September 2024. Clinical interviews were performed by qualified experts based on the International Classification of Diseases, 11th Edition (ICD-11) for inpatients at the Department of Mental Health, Shanxi Bethune Hospital. Eligible participants were enrolled following predefined inclusion and exclusion criteria and completed standardized questionnaire assessments. Inclusion criteria were as follows: 1)Inpatients admitted to the Department of Mental Health; 2) No history of muscle-relaxant-free electroconvulsive therapy within the prior month; 3) Cognitive capacity to understand assessment materials. Exclusion criteria comprised: 1) Diagnosed cognitive impairment; 2) Presence of severe unstable physical illnesses at the time of enrollment.

The initial sample consisted of 519 participants, of which 411 valid questionnaires were retained after excluding cases with missing values. The final sample included 124 males and 287 females, aged 9 to 81 years (mean = 36.91, SD = 19.96). The duration of hospitalization ranged from 1 to 37 days (mean = 13.05, SD = 4.2; median = 14 days, interquartile range = 11–15days). Diagnostic distributions were as follows: 181 patients (44%) with depressive disorders, 135 (33%) with anxiety or fear-related disorders, 24 (6%) with psychotic disorders, 25 (6%) with bipolar disorder, and 46 (11%) with other conditions including dissociative disorders, somatic symptom disorders, and eating disorders.

The study was approved by the Ethics Committee of Peking University. Informed consent was obtained from all participants or their legal guardians, with parents or legal guardians specifically authorizing participation for patients under 16 years of age. The research adhered to the ethical principles outlined in the 2013 revised Declaration of Helsinki (22), maintaining rigorous ethical standards throughout the study period. Written permission was obtained from the participating institutions. All data were anonymized to protect participant privacy, and individuals were explicitly informed of their right to withdraw from the study at any time without penalty.

### 2.2 Measures

#### 2.2.1 Athens Insomnia Scale-8

The Athens Insomnia Scale-8 (AIS-8) was originally developed to measure insomnia severity based on ICD-10 criteria (11). For this study, the Chinese version was used in this study. for inpatient use. Each item uses a 4-point response scale (0 = “no problem” to 3 = “serious problem”), with a total score range of 0–24. Higher scores indicate more severe insomnia. The Cronbach’s α coefficient for AIS-8 in this sample was 0.84, demonstrating good internal consistency.

#### 2.2.2 Patient Health Questionnaire-9

The Patient Health Questionnaire-9 (PHQ-9) assesses depressive symptoms based on DSM-IV criteria, consisting of nine items. Each item employs a Likert-type scale (0–3), where 0 = “Not at all, ” 1 = “several days, ” 2 = “more than 1 week, ” and 3 = “nearly every day.” Previous research has validated its reliability and validity in Chinese populations (23, 24). In this study, the PHQ-9 exhibited a Cronbach’s α of 0.88, indicating strong internal consistency.

#### 2.2.3 Generalized Anxiety Disorder-7

Developed by Spitzer and Kroenke (25), the Generalized Anxiety Disorder-7 (GAD-7) is a self-administered scale for measuring anxiety severity in clinical and research settings. The seven-item scale asks participants to rate how often they experienced each symptom over the past two weeks, using a 0–3 scoring system (0 = “not at all” to 3 = “nearly every day”). Total scores range from 0–21. The instrument has demonstrated good internal consistency in Chinese samples (8). In this study, the GAD-7 had a Cronbach’s α of 0.87, confirming its reliability.

### 2.3 Statistical analysis

Statistical analyses were performed using SPSS 20 and Mplus 8.3.0.1. Specifically, SPSS 20 was employed for reliability testing, exploratory factor analysis (EFA), and criterion validity assessment of the Chinese AIS-8, while Mplus 8.3.0.1 was utilized for confirmatory factor analysis (CFA) and measurement invariance testing across demographic subgroups. The full sample was randomly partitioned into two subsets: one for EFA and the other for CFA.

Prior to EFA, the Kaiser-Meyer-Olkin (KMO) measure of sampling adequacy and Bartlett’s test of sphericity were computed to assess factorability. Values of KMO > 0.5 and significant Bartlett’s test (p < 0.05) indicated suitability for factor analysis (7). EFA was conducted using robust maximum likelihood estimation (MLR) with oblique Geomin rotation to extract the underlying factor structure.

For confirmatory factor analysis (CFA), model fit was evaluated using the following indices (26): Satorra-Bentler chi-square (SB-χ²), comparative fit index (CFI), Tucker-Lewis Index (TLI), and root mean square error of approximation (RMSEA). Given the sensitivity of the SB-χ² statistic to sample size (27), the χ²/df ratio (CMIN/DF) was also considered, with values <3 indicating adequate fit. CFI and TLI values >0.90 were deemed indicative of good fit, while RMSEA values <0.08 were considered acceptable.

Subsequent to the CFA, measurement invariance across age, gender, and occupational groups was evaluated via multigroup confirmatory factor analysis (MG-CFA) using a hierarchy of nested models: configural invariance (unconstrained parameter estimation), metric invariance (constrained factor loadings), scalar invariance (additional constraints on intercepts), and strict invariance (further constraints on residual variances). Model fit across nested models was evaluated using criteria of ΔSB-χ² > 0.05, ΔCFI < 0.01, and ΔRMSEA < 0.015, as recommended in previous studies (27, 28). Given the sample size sensitivity of the ΔSB-χ² statistic, ΔCFI and ΔRMSEA were prioritized as more robust indicators of model invariance.

Cronbach’s α coefficient, with values >0.7 traditionally regarded as indicating acceptable internal consistency (29, 30). Given prior evidence that the PHQ-9 and GAD-7 are positively correlated with the AIS, convergent validity was assessed by examining correlations between the AIS and these measures. A valid AIS was expected to demonstrate positive associations with both the PHQ-9 and GAD-7.

## 3 Results

### 3.1 Demographic features

Age groups were divided at 16 years based on legal definitions of adolescence and adulthood in China. The final sample of 411 inpatients comprised 110 individuals (26.8%) aged 16 years or younger and 301 (73.2%) aged 17 years and older. Duration of hospitalization ranged from 1 to 37 days (mean = 13.05, SD = 4.21). Marital status distributions were as follows: single (n = 181, 44%), married (n = 217, 52.8%), and widowed/divorced (n = 13, 3.2%). In terms of occupational status, 148 participants (36%) were students, while 263 (64%) were non-students.

### 3.2 Item analysis

Participants were divided into high and low score groups based on total scores, with the top 25% categorized as the high-score group and the bottom 25% as the low-score group. An independent samples t-test revealed significant differences between the two groups (P <.001), indicating that each item effectively discriminates between high and low scorers. As presented in **Table 1**, internal homogeneity was supported by item-total correlations (.411-.812), corrected item-total correlations (.223-.730), and Cronbach’s alpha values if each item were deleted (.797-.866). Although deleting Item 8 resulted in a Cronbach’s alpha of.87, which exceeded the scale’s overall internal consistency of.84, Item 8 was retained due to its minimal impact on reliability and its strong discriminatory power between high and low score groups.

**Table 1:** Item analysis for AIS in the Chinese inpatient populations.

Items of AIS	Item-total correlations	Corrected item-total correlations	Cronbach’s alpha if item deleted
Sleep induction	.714**	.609	.814
Awakenings during the night	.753**	.654	.808
Final awakening earlier	.736**	.625	.811
Total sleep duration	.812**	.730	.797
Sleep quality	.795**	.713	.800
Daytime wellbeing	.635**	.516	.825
Daytime functioning capacity	.653**	.539	.823
Daytime sleepiness	.411**	.223	.866

** represents P<.01.

### 3.3 Exploratory factor analysis

The participants were randomized into two groups, with a total of 206 participants included in the EFA. Given the divergent findings regarding the factor structure of the AIS, an exploratory factor analysis (EFA) was conducted to examine its latent structure in Chinese inpatient populations. A total of 411 participants were randomly allocated, with the final EFA sample consisting of 206 individuals. As shown in **Table 2**, the EFA and CFA subsamples were comparable in gender, age, and clinical diagnosis. The analysis was performed using oblique GEOMIN rotation and robust maximum likelihood estimation (MLR). Guided by the Kaiser criterion (eigenvalues > 1) and inspection of the scree plot, a two-factor solution was retained. As reported in **Table 3**, the two factors had eigenvalues of 3.772 and 1.302, respectively, accounting for 63.43% of the total variance. The rotated factor loadings and communalities are presented in **Table 4**. Items 1–5 loaded primarily on Factor 1, with loadings ranging from 0.572 to 0.849, whereas Items 6–8 loaded primarily on Factor 2, with loadings ranging from 0.412 to 0.853. Cross-loadings were generally low, indicating a relatively clear factor structure, and communalities ranged from 0.150 to 0.730. As shown in **Table 5**, the correlation between the two factors was 0.571, supporting the use of oblique rotation. Based on the item content, the two factors were labeled “nocturnal sleep problems” and “daytime dysfunction.

**Table 2:** Demographic and clinical comparability of the EFA and CFA samples.

Variable	EFA sample (n = 206)	CFA sample (n = 205)
Gender, n (%)		
Male	66 (32.0%)	58 (28.3%)
Female	140 (68.0%)	147 (71.7%)
Age, years		
Mean ± SD	36.73 ± 20.21	37.09 ± 19.75
Median	36	37
Range	9–76	10–81
Age group (16-year cutoff), n (%)		
≤16 years	59 (28.6%)	51 (24.9%)
>16 years	147 (71.4%)	154 (75.1%)
Broad diagnostic category, n (%)		
Depressive disorders (6A7)	93 (45.1%)	88 (42.9%)
Anxiety/fear-related disorders (6B)	68 (33.0%)	67 (32.7%)
Bipolar disorders (6A6)	13 (6.3%)	12 (5.9%)
Psychotic disorders (6A2)	12 (5.8%)	12 (5.9%)
Bodily distress disorders (6C2)	6 (2.9%)	10 (4.9%)
Other disorders	14 (6.8%)	16 (7.8%)

The final sample included 411 inpatients after excluding cases with missing data. Diagnostic categories were coded according to the ICD-11 diagnostic grouping used in the original dataset.

**Table 3:** Eigenvalues.

Factor number	Eigenvalue
1	3.772
2	1.302
3	0.754
4	0.657
5	0.499
6	0.422
7	0.355
8	0.240

**Table 4:** Factor loadings and communalities.

Item	Factor 1	Factor 2	Communality
AIS1	0.772	-0.096	0.521
AIS2	0.786	-0.142	0.510
AIS3	0.572	0.095	0.398
AIS4	0.849	0.010	0.730
AIS5	0.809	0.014	0.668
AIS6	0.006	0.648	0.424
AIS7	0.002	0.853	0.729
AIS8	-0.045	0.412	0.150

**Table 5:** Factor correlation matrix.

Factor	Factor 1	Factor 2
Factor 1	1.000	0.571
Factor 2	0.571	1.000

### 3.4 Confirmatory factor analysis

The remaining half of the sample (205) was subjected to CFA using a two-factor-correlated model specifying nocturnal sleep and daytime dysfunction factors. We tested three competing models where results were illustrated in **Table 6**. two-factor Results showed that two factor models achieved adequate fit. Although the three-factor model demonstrated acceptable overall fit, it was not retained because the estimated correlation between two latent factors exceeded 1.00, indicating an improper and inadmissible solution. Moreover, Considering the relative low factor loadings of AIS 8, we deleted it and ran the model. It indicated a worse fit than the original two-factor models. **Tables 7**, **8** suggested the factor loadings of the two-factor models with and without AIS-8.

**Table 6:** Two-factor the model fit of four models of CFA.

Model	χ²	df	χ²/df	RMSEA	TLI	CFI
One-factor model (8 items: AIS1-8)	100.37	20	5.02	0.14	0.825	0.875
Two-factor model (with AIS8) (F1:1-5, F2:6-8)	25.042	19	1.32	0.039	0.986	0.991
Two-factor model (without AIS8) (F1:1-5, F2:6-7)	18.581	13	1.43	0.046	0.985	0.991

**Table 7:** The standardized factor loadings of the two-factor model with AIS-8.

Item	Factor	Standardized loading
AIS1	F1	0.692
AIS2	F1	0.827
AIS3	F1	0.77
AIS4	F1	0.876
AIS5	F1	0.85
AIS6	F2	0.807
AIS7	F2	0.787
AIS8	F2	0.344

**Table 8:** The standardized factor loadings of the two-factor model without AIS-8.

Item	Factor	Standardized loading
AIS1	F1	0.691
AIS2	F1	0.827
AIS3	F1	0.772
AIS4	F1	0.876
AIS5	F1	0.849
AIS6	F2	0.844
AIS7	F2	0.748

Using the Maximum Likelihood estimator, multigroup CFA was performed based on the established two-factor model to test the measurement invariance of the AIS across subgroups of Chinese inpatients. The configural invariance model was first specified as the baseline, followed by sequential testing of three increasingly restrictive models: metric invariance (constrained factor loadings), scalar invariance (additional constraints on intercepts), and strict invariance (constrained residual variances). We conducted measurement invariance for age (16 or below/above 16), gender (male and female), occupation (students and non-students) and marital status (single/marriage/widowed and divorced) subgroup. It is noteworthy that when conducted measurement invariance for marital status, divorced/widowed subgroup were added in the single group due to relatively small sample size.

As shown in **Table 9**, the metric invariance, scalar invariance, and strict invariance models demonstrated good fit for gender (ΔCFI < 0.01, ΔRMSEA < 0.015). However, for age, marital status and occupation groups, only weak metric invariance was achieved (ΔCFI < 0.01, ΔRMSEA < 0.015).

**Table 9:** Measurement invariance across gender, age, marital status, and occupation.

Group	Model	χ²	df	χ²/df	CFI	RMSEA	ΔCFI	ΔRMSEA
Gender	Configural	43.256	38	1.138	0.993	0.037		
	Metric	51.737	44	1.176	0.990	0.041	0.003	0.004
	Scalar	59.286	50	1.186	0.988	0.043	0.002	0.002
	Strict	70.511	58	1.216	0.984	0.046	0.004	0.003
Age (≤16 vs. >16)	Configural	39.124	38	1.030	0.999	0.017		
	Metric	48.239	44	1.096	0.995	0.031	0.004	0.014
	Scalar	64.840	50	1.297	0.981	0.054	0.014	0.023
	Strict	99.399	58	1.714	0.948	0.083	0.033	0.029
Marital status	Configural	30.276	38	0.797	1.000	0.000		
	Metric	35.714	44	0.812	1.000	0.000	0.000	0.000
	Scalar	77.913	50	1.558	0.966	0.074	0.034	0.074
	Strict	137.554	58	2.372	0.902	0.116	0.064	0.042
Occupation	Configural	33.312	38	0.877	1.000	0.000		
	Metric	40.601	44	0.923	1.000	0.000	0.000	0.000
	Scalar	88.332	50	1.767	0.953	0.086	0.047	0.086
	Strict	141.987	58	2.448	0.898	0.119	0.055	0.033

### 3.5 Internal consistency

Internal consistency reliability of the AIS in Chinese inpatient populations was evaluated using Cronbach’s alpha. The AIS-8 demonstrated a Cronbach’s alpha of 0.84, exceeding the acceptable threshold of 0.70. Similarly, the PHQ-9 and GAD-7 showed Cronbach’s alpha coefficients of 0.88 and 0.87, respectively, indicating good internal consistency. These findings suggest that the AIS-8, PHQ-9, and GAD-7 all demonstrated satisfactory reliability in Chinese psychiatric inpatients.

### 3.6 Convergent validity

To assess the convergent validity of the AIS-8, Pearson’s correlation coefficients were computed between the AIS-8 and PHQ-9, as well as between the AIS-8 and GAD-7, using the total inpatient sample. All scales exhibited score distributions that covered nearly the entire possible range. Skewness values (ranging from −0.70 to −0.21) and kurtosis values (ranging from −0.54 to −0.15) indicated that all scales approximated a normal distribution (31). Also, results revealed positive correlations between the AIS-8 and both the PHQ-9 (r = 0.50) and GAD-7 (r=0.44), reflecting moderate effect sizes consistent with theoretical expectations.

## 4 Discussion

This study investigated the psychometric properties of the AIS-8 in Chinese inpatient populations, addressing a critical gap in the validation of AIS within inpatient psychiatric samples. Findings indicated that the AIS-8 is reliable and preliminarily valid for measuring insomnia severity, consistent with prior cross-population studies across diverse age groups (7, 21, 32). In the EFA, a two-factor structure two-factor was established, differing from the original single-factor model and the Spanish version (11, 19). The extracted factors, “nighttime sleep problems” and “daytime dysfunction, ” align with findings in Japanese, Mexican, and previous Chinese populations (7, 13, 14).

Regarding the CFA results, two-factor model two-factor comprising ‘nighttime sleep problems’ and ‘daytime dysfunction’ exhibited acceptable fit indices.

, confirming good model adequacy. Although Item 8 (“daytime sleepiness”) showed a low factor loading (0.34), its removal deteriorated model fit, necessitating its retention. This finding aligns with a Japanese study in chronic pain patients, where Item 8 also had a low factor loading (0.2) (32), and its exclusion similarly worsened model fit. Converging evidence thus suggests that Item 8, despite low factor loading, contributes to optimal model fit and is integral to insomnia measurement.

Early studies (13, 14, 19) rarely investigated measurement invariance, but subsequent research (7, 17, 18, 33, 34) increasingly examined invariance across demographics like gender and age. Consistent with prior findings (7, 17, 33), gender in this sample achieved strict invariance. Age, however, failed to meet strict invariance criteria (7, 33), only reaching weak invariance rather than scalar (strong) invariance. This discrepancy may stem from age range differences: prior studies (7) included participants aged 26–100, whereas our sample spanned childhood to adolescence. Occupational grouping (students vs. non-students), which overlapped with age, similarly showed weak invariance. Marital status (single/married) also achieved weak invariance, indicating marital impact on AIS application in inpatients.

The present study demonstrated good reliability of the AIS in Chinese inpatients, consistent with cross-national findings across diverse age groups (15, 32). These results, alongside accumulating evidence, provide preliminary support for the AIS as a useful measure of insomnia severity. Furthermore, convergent validity was established through significant positive correlations between the AIS and both PHQ-9 and GAD-7, aligning with empirical evidence linking insomnia to anxiety and depression (6–10).

This study has several limitations that warrant attention. First, discriminant validity was not tested, which may limit the scale’s ability to distinguish insomnia from other constructs. Second, the sample size was insufficient to support robust multigroup analysis, and the excessively broad age range introduced substantial sample heterogeneity. Additionally, the study’s single-center design and lack of inclusion of ethnic minority groups restrict its generalizability to diverse Chinese populations. Third, the absence of clinical insomnia diagnoses impeded the exploration of optimal cutoff values for Chinese inpatients, while the lack of discriminant validity testing precluded the differentiation of insomnia from other sleep disorders (e.g., sleep apnea). Moreover, the diverse psychiatric diagnoses introduced sample heterogeneity. In addition, although convergent validity was supported by significant positive correlations with PHQ-9 and GAD-7, these scales assess depressive and anxiety symptoms rather than sleep-specific constructs. The absence of a dedicated sleep-related instrument means the evidence for convergent validity remains relatively weak. This limitation should be considered when interpreting the present findings.

In summary, the present study evaluated the psychometric properties of the AIS-8 in a diverse sample of Chinese inpatients, yielding evidence of acceptable reliability and validity. Specifically, a two-factor structure, consisting of “nighttime sleep problems” and “daytime dysfunction”, was empirically supported. This distinct two-factor structure may provide valuable insights for the development of targeted interventions, such as cognitive behavioral therapy tailored to sleep initiation difficulties versus strategies focused on daytime fatigue management. With regard to measurement invariance, strict invariance was confirmed across gender, while weak invariance was observed across age, occupation, and marital status. Collectively, these findings offer robust and reliable evidence that the AIS-8 is a practically useful and psychometrically sound instrument for assessing insomnia severity among Chinese psychiatric inpatients.

## Data Availability

The raw data supporting the conclusions of this article will be made available by the authors, without undue reservation.
